# High prevalence of *Trichomonas gallinae* in wild columbids across western and southern Europe

**DOI:** 10.1186/s13071-017-2170-0

**Published:** 2017-05-18

**Authors:** Melanie Marx, Gerald Reiner, Hermann Willems, Gregorio Rocha, Klaus Hillerich, Juan F. Masello, Sylvia L. Mayr, Sarah Moussa, Jenny C. Dunn, Rebecca C. Thomas, Simon J. Goodman, Keith C. Hamer, Benjamin Metzger, Jacopo G. Cecere, Fernando Spina, Steffen Koschkar, Luciano Calderón, Tanja Romeike, Petra Quillfeldt

**Affiliations:** 1 Department of Animal Ecology & Systematics, Justus-Liebig-University Giessen, Heinrich-Buff-Ring 38, 35392 Giessen, Germany; 2 Department of Clinical Veterinary Sciences, Justus-Liebig-University, Frankfurter Strasse 112, 35392 Giessen, Germany; 3 Department of Agro-forestry Engineering, University of Extremadura, Avda. Virgen del Puerto 2, 10600 Plasencia, Cáceres Spain; 4 Röntgenstraße 7, 64823 Groß-Umstadt, Germany; 5 School of Life Sciences, University of Lincoln, Joseph Banks Laboraties, Lincoln, LN6 7TS UK; 6 School of Biology, Irene Manton Building, University of Leeds, Leeds, LS2 9JT UK; 7 BirdLife Malta, Xemxija Waterfront Apartments, Flat 1/2, Triq Is-Simar, Xemxija, St Paul’s Bay, SPB 9025 Malta; 8 ISPRA, Institute for Environmental Protection and Research, Via Ca’ Fornacetta 9, 40064 Ozzano Dell’Emilia, BO Italy; 9 Dorfstraße 11, 02923, Biehain, Germany

**Keywords:** *Trichomonas gallinae*, Columbiformes, Stock dove, Phylogenetic analysis, Genetic lineage, Pathogen

## Abstract

**Background:**

Avian trichomonosis is known as a widespread disease in columbids and passerines, and recent findings have highlighted the pathogenic character of some lineages found in wild birds. Trichomonosis can affect wild bird populations including endangered species, as has been shown for Mauritian pink pigeons *Nesoenas mayeri* in Mauritius and suggested for European turtle doves *Streptopelia turtur* in the UK. However, the disease trichomonosis is caused only by pathogenic lineages of the parasite *Trichomonas gallinae*. Therefore, understanding the prevalence and distribution of both potentially pathogenic and non-pathogenic *T. gallinae* lineages in turtle doves and other columbids across Europe is relevant to estimate the potential impact of the disease on a continental scale.

**Results:**

We examined 281 samples from four wild columbid species for *Trichomonas* infection and determined the genetic lineages. The overall prevalence was 74%. There were significant differences between the species (*P* = 0.007). The highest prevalence was found in stock doves *Columba oenas* (86%, *n* = 79) followed by wood pigeons *Columba palumbus* (70%, *n* = 61) and turtle doves (67%, *n* = 65), while three of five collared doves *Streptopelia decaocto* (60%) were infected. We found seven different lineages, including four lineages present in columbids in the UK, one lineage already described from Spain and three new lineages, one of those found in a single turtle dove migrating through Italy and another one found in a breeding stock dove. Stock doves from Germany and collared doves from Malta were infected with a potentially pathogenic lineage (lineage A/B), which is known to cause lesions and mortality in columbids, raptors and finches.

**Conclusions:**

Generally, turtle doves showed high prevalence of *Trichomonas* infection. Furthermore, the potentially pathogenic lineage A/B (or genotype B according to previous literature) was found in a recovering stock dove population. Both findings are worrying for these columbid species due to the occasional epidemic character of trichomonosis, which can have severe negative effects on populations.

**Electronic supplementary material:**

The online version of this article (doi:10.1186/s13071-017-2170-0) contains supplementary material, which is available to authorized users.

## Background

The protozoan parasite *Trichomonas gallinae* infects captive and wild bird species across the world and can lead to the avian disease called trichomonosis. Due to its widespread occurrence and sometimes pathogenic character, it is thought to play a role in the regulation of wild bird populations [1–5]. Bird migrations can contribute to the spread of *Trichomonas*. For instance, the drastic decline observed in greenfinch *Chloris chloris* and chaffinch *Fringilla coelebs* in the UK and Fennoscandia as well as the increased mortality of finches in Germany and Austria was linked to spread of the parasite by bird species during migration [4, 6–9].

Birds belonging to the Columbiformes are the main hosts of *Trichomonas gallinae* [1, 3] and rock pigeons *Columba livia* are thought to be the predominant transmitters of *T. gallinae* worldwide [1]. *Trichomonas* parasites also occur in wild columbid species around the world [10–14]. In Europe, columbids from wild populations in Spain and the UK were surveyed recently. Trichomonosis has been detected in wild European columbid species including collared doves *Streptopelia decaocto*, stock doves *Columba oenas*, wood pigeons *Columba palumbus* and European turtle doves *Streptopelia turtur* (hereafter turtle doves) [3, 12, 15–17].

Turtle doves, stock doves and wood pigeons are migratory birds, which can favour the spread of *Trichomonas* parasites. Turtle doves display three main migration flyways, a western, central and eastern route between Europe and sub-Saharan Africa [18]. Stock doves and wood pigeons from European countries mainly use the western migratory flyway to France and Iberia [19, 20]. As pathogens can be spread by migratory birds, those species might be more vulnerable to parasite exposure [6].

Within the Columbiformes, parasite transmission can occur at shared feeding and drinking sites within and among species and from adults to nestlings while crop milk feeding [1, 21]. Epidemic trichomonosis in finches showed a relatively low host specificity of the parasite [22], with parasite spill-over probably occurring at feeding and watering stations shared by different bird species [3, 23]. If birds are infected by *Trichomonas*, they can develop necrotic lesions in the crop and oropharynx (i.e. trichomonosis), which can lead to death by starvation and suffocation [1]. Moreover, not only the upper digestive tract can be affected, but also less commonly the liver, air sacs as well as parts of the cranium [1]. Nevertheless, there are individual differences in disease response and not all infected birds show clinical signs, because the virulence of the parasite varies among different lineages [4, 14, 24]. For instance, it might happen a bird shows clinical signs of the disease, such as caseous lesions, but recovers after some days [1, 25]. On the other hand, there are highly pathogenic lineages, which weaken birds severely, cause lesions to develop in oropharynx and liver, and lead to death in almost any case [25]. Furthermore, outbreaks can occur even when food shortage or stress is present because birds get more vulnerable to parasite exposure and infection due to limited food sources and crowding with probably infected birds [3]. However, Stabler [26] also showed the possible immunisation with less pathogenic *Trichomonas* lineages in birds. Nevertheless, as reported in finches, the most pathogenic lineages can heavily affect population sizes [6]. Thus, trichomonosis might represent an additional threat to endangered bird species, such as Mauritian pink pigeons *Nesoenas mayeri* in Mauritius [10] or turtle doves in Europe [27, 28]. Likewise, recovering stock dove populations, which showed population declines in the 20th century due to a lack of adequate breeding sites [19, 29], may be threatened again in the future by this emerging infectious disease due to their migratory character and the low host specificity of *Trichomonas* parasites.

Here we examined samples from four different columbid species from Germany, Spain, Italy and Malta. Spanish and UK turtle dove populations are connected through the western European flyway, and turtle doves from western Germany also migrate through Spain [18]. Furthermore, Italian and Maltese turtle doves follow a central/eastern flyway [18]. The aims of the present study were to (i) test whether turtle doves migrating through Italy and Malta, along the central/eastern flyway [18], have similar prevalence and genetic lineages of *Trichomonas* as birds along the western flyway; (ii) obtain baseline data on prevalence and genetic lineages of *Trichomonas* in a recovering population of stock doves in Germany; and (iii) provide information from a larger sample of turtle doves, collared doves (sampled in Malta), stock doves and wood pigeons migrating along the western flyway (sampled in Spain and Germany), with a focus on the potentially pathogenic lineages (in Lennon et al. [12]: lineages 3 and 4, which belong to the genotype B [14]), which are known to cause gross lesions and mortality in columbids, raptors and finches [12, 14].

## Methods

### Species and study sites

A total of 281 columbids from four different countries (Germany, *n* = 180; Spain, *n* = 45; Malta, *n* = 36; and Italy, *n* = 20) were sampled for *Trichomonas* infections, with the main focus on stock doves and wood pigeons from Germany and turtle doves on migration through Malta and Italy as well as from a breeding population in Spain. We also took samples from Spanish and Maltese collared doves. 80% of sampled birds came from Western Europe, and 20% of birds came from Central Europe. Samples from stock doves included breeding adults (*n* = 33), chicks (*n* = 58) and one first-year bird. Considering stock dove chicks, we had 47 siblings out of 23 nests. Of those nests, 22 included two siblings, and one nest had three siblings. Additionally, we sampled adult and first-year turtle doves at the end of the breeding season in Extremadura, Spain (39°51′42″N, -6°6′37″E). Other turtle doves were sampled during the non-breeding season, birds during spring migration on Comino, Malta (36°0′36″N, 14°20′8″E) and Italy (on the island of Ventotene: 40°47′54″N, 13°25′55″E) (Table 1). Unfortunately, for almost half of the turtle doves, we could not determine the age, thus they were classified as “unknown” age.

**Table 1:** *Trichomonas* spp. prevalence found in four columbid species at different European study sites with the use of PCR analysis. The prevalence was calculated according to PCR results

Species	No. of samples	Place (*n*)	Status (*n*)	Sample (*n*)	No. positive	No. negative	Prevalence [%]	No. of sequences
Stock dove	92	Hesse, Germany (90) Brandenburg, Germany (2)	Adult (33) First year (1) Chick (58)	Swab (92)	79	13	86	16
Wood pigeon	87	Bavaria, Germany (50) North Rhine-Westphalia, Germany (24) Lower Saxony, Germany (8) Thuringia, Germany (3) Hesse, Germany (1) Unknown, Germany (1)	Adult (87)	Tissue (87)^a^	61	26	70	23
Collared dove	5	Monfrague, Spain (3) Comino, Malta (2)	Adult (3) Second year (1) Unknown (1)	Swab (5)	3	2	60	2
Turtle dove	97	Monfrague, Spain (42) Comino, Malta (34) Ventotene, Italy (20) Hesse, Germany (1)	Adult (22) Juvenile (23) Second year (2) Unknown (50)	Swab (70) Tissue (27) All	65 0 65	5 27 32	93 0 67	43 0 43
Total	281			Swab (167) Tissue (114) All	154 61 208	13 53 73	93 54 74	61 23 84

^a^One of the wood pigeons (sample WP4) had yellow plaque

### Sampling and parasite culture

The Veterinary Department of the Justus-Liebig University Giessen obtained dead adult wood pigeons from hunting managers from different sites in Germany. Therefore, they constitute a random sample of the population, as they had not died from natural causes. To obtain a tissue sample, we opened the oropharynges of 87 defrosted birds from outside with a sterile scalpel and cut a small tissue sample from the oropharyngeal tract. While we extracted the tissue sample, we also checked the pigeon for lesions. Only one wood pigeon had a yellow plaque in the throat. Furthermore, we obtained 27 samples of oropharyngeal tissue from migrating turtle doves hunted in Malta. The tissue samples were kept at -18 °C until DNA extraction. Since we could not investigate the entire bird, we cannot tell, whether these Maltese birds had lesions in their oesophagus.

Oral swabs were taken from oropharynx and crop after visual inspection of 41 freshly hunted birds in Spain and 126 live birds at all other sites with a dry, sterile cotton tip. No lesions were detected, and swabs were inoculated individually in a *Trichomonas* selective culture medium (OXOID Deutschland GmbH, Wesel, Germany) (*n* = 20) or an InPouch TF culture kit (BioMed Diagnostics, Oregon, USA) (*n* = 147). Both media have similar detection sensitivities for *Trichomonas* parasites and have been used successfully in Finland [30]. The samples were incubated at 37 °C for five to seven days, giving any protozoan parasites sufficient time to multiply [31]. Samples were centrifuged for 5 min at 1,000× *g*, the supernatant was discarded, and the pellet was re-suspended in 1 ml of phosphate-buffered saline (PBS). The samples were centrifuged again for 5 min at 1,000× *g* the pellet was re-suspended in five drops of PBS (approximately 100 μl) and kept at -18 °C until DNA extraction.

### DNA extraction

DNA from tissue samples was extracted using the DNeasy blood and tissue kit (Qiagen, Hilden, Germany) following the manufacturer’s protocol. For final elution, we used 60 μl of ddH_2_O to increase DNA concentration in the eluate. *T. gallinae* swab samples were centrifuged for 5 min at 13,000× *g*. The supernatant was discarded, and DNA was extracted as described by Mégia-Palma et al. 2013 [32]. The DNA was re-suspended in 30 μl of ddH_2_O. The DNA concentration of all samples was measured with a ThermoScientific Nanodrop 2000 micro-volume UV-VIS spectrometer. For samples with a concentration above 70 ng/μl, the sample was diluted with ddH_2_O to a final concentration of at least 20 ng/μl.

### ITS1-5.8S-ITS2 PCR amplification and gel electrophoresis

For infection detection, we amplified the highly conserved ITS1-5.8S-ITS2 ribosomal region of the *T. gallinae* genome [33] with the primers TFR1 (5′-TGC TTC AGT TCA GCG GGT CTT CC-3′) and TFR2 (5′-CGG TAG GTG AAC CTG CCG TTG G-3′) [34], which produce an expected product of 400 bp [4]. We applied two different reagents and reaction volumes for (i) the samples from Malta, and (ii) all other samples due to different laboratory environments, caused by the opportunity to use a new laboratory when Maltese samples became available. For Maltese samples, we used 20 μl reaction volume per sample, including 17.5 μl Dream*Taq* PCR Mastermix (2×) (Thermo Scientific, Germany). The mastermix contained DreamTaq DNA Polymerase, 2× Dream*Taq* buffer, 0.4 mM of each dNTP and 4 mM MgCl_2_, TFR1 and TFR2 (both 20 μM) and ddH_2_O. Furthermore, we added 2.5 μl of template DNA.

For polymerase chain reactions (PCR) of the remaining samples, we used 10 μl reaction volume per sample, including 5 μl of 2× MM Mastermix (Qiagen) (with HotStar*Taq* DNA Polymerase, PCR buffer containing 3 mM MgCl_2_, 400 μM of each dNTP), 2 μl H_2_O, 1 μl loading dye (containing 0.3% Orange G and 25% Saccharose) and 1 μl primer mix, containing 20 μM of both, TFR1 and TFR2 and added 1 μl of template DNA.

The reactions of the Maltese samples were conducted on a peqstar 96Q Real-Time PCR cycler (PEQLAB Biotechnologie GmbH, Erlangen, Germany). PCR reactions of all other samples were conducted on a Biometra TPersonal Thermocycler (Biometra, Göttingen, Germany). All PCR reactions were run with a negative control. We applied following cycling conditions for all PCR reactions: polymerase activation at 95 °C for 15 min, followed by 35 cycles with a denaturation at 94 °C for 30 s, annealing at 60 °C for 90 s and extension at 72 °C for 60 s. Final extension was set to 72 °C for 10 min.

We used gel electrophoresis to visualise PCR products, and positive samples were sequenced at either SEQLAB (Sequence Laboratories Göttingen, Germany) (Maltese samples) or the Konrad Lorenz Institute of Ethology (University of Veterinary Medicine Vienna, Austria) (all other samples). In total, two PCR products from Maltese collared doves, 34 PCR products from stock doves, 39 from wood pigeons and 49 from turtle doves were sent for sequencing. We assumed that most of our samples came from unrelated individuals, except for two pairs of siblings from two nests of stock doves that were included to determine whether they were infected by the same *Trichomonas* lineage.

Furthermore, we applied a Chi-square test using R 3.2.4 [35] to check for differences in prevalence between columbid species. Due to the small sample size of collared doves, those were excluded from statistical analyses.

### Fe-hydrogenase PCR amplification and capillary electrophoresis

We performed PCR reactions for all samples that were tested positive and used in the phylogenetic analysis for ITS1/5.8S/ITS2 We used the primers TrichhydFOR (5′-GTT TGG GAT GGC CTC AGA AT-3′) and TrichhydREV (5′-AGC CGA AGA TGT TGT CGA AT-3′) [22]. For Fe-hydrogenase gene PCR amplifications the Multiplex PCR Plus Kit (Qiagen) was used. That leads to higher PCR outputs, when samples were stored in PBS because PBS inhibits PCR reactions due to high chloride concentrations [36].

For polymerase chain reactions, we used 15 μl reaction volume per sample, including 7.5 μl of 2× Qiagen Multiplex PCR Mastermix (Qiagen) (with HotStar*Taq* DNA Polymerase, PCR buffer containing 6 mM MgCl_2_, and ultrapure quality of dNTPs), 2 μl H_2_O, 0.75 μl of each primer (10 μM concentration) and added 4 μl of template DNA.

PCR reactions were conducted on a Biometra TONE Thermocycler (Biometra, Göttingen, Germany). All PCR reactions were run with a negative and a positive control. The positive control originated from a British greenfinch in 2007 [37]. We applied following cycling conditions for all PCR reactions: polymerase activation at 95 °C for 5 min, followed by 35 cycles with a denaturation at 95 °C for 30 s, annealing at 57 °C for 90 s and extension at 72 °C for 90 s. Final extension was set to 72 °C for 5 min.

We used capillary electrophoresis (QIAxcel Advanced, Qiagen, Switzerland) to visualise PCR products and positive samples were sequenced at SEQLAB (Sequence Laboratories Göttingen, Germany). In total, six PCR products were sent for sequencing. Five came from turtle doves, and one originated from a collared dove.

### Phylogenetic analysis of the ITS1-5.8S-ITS2 region

Forward and reverse sequences were assembled and trimmed with CLC Main Workbench 7.6.1 (CLC bio, Qiagen). With NCBI Blast [38] we checked every sequence for its closest GenBank match and downloaded these as reference sequences (see Additional file 1: Table S1 for an overview about the percentage of identity to the closest GenBank match). Additionally, we downloaded the closest GenBank matches, obtained for columbids by Lennon et al. [12]. This enables the direct comparison of *Trichomonas* lineages occurring in stock doves, wood pigeons and turtle doves from the UK to Germany, Italy and Spain. *Tritrichomonas foetus* (GenBank accession number DQ243911.1 [39]) was used as outgroup for phylogenetic analysis following previous studies [12]. We aligned all sequences using BioEdit [40]. The nucleotide substitution model that best fitted our alignment was determined with MEGA 6.0 [41] using Bayesian Information Criterion scores. The phylogenetic tree was also inferred with MEGA 6.0 [41] using the Maximum Likelihood algorithm and employing the Tamura 3 substitution model with invariant sites. Node support was assessed after 1000 bootstrap pseudo-replicates. The GenBank accession numbers of analysed sequences are given in Additional file 2: Table S3.

### Phylogenetic analysis of the Fe-hydrogenase gene

Forward and reverse sequences were assembled and trimmed with CLC Main Workbench 7.6.1 (CLC bio, QIAGEN). With NCBI Blast [38] we checked every sequence for its closest GenBank match and downloaded these as reference sequences (see Additional file 3: Table S2 for an overview about the percentage of identity to the closest GenBank match). We also downloaded the reference sequences used in Chi et al. 2013 [42] to specify the sub-lineages of *Trichomonas gallinae. Trichomonas vaginalis* (GenBank accession number: XM_001310179.1 [43]) was used as the outgroup for phylogenetic analysis following previous studies [22]. We aligned all sequences using BioEdit [40]. The phylogenetic tree was inferred with MEGA 6.0 [41] using the Maximum Likelihood algorithm and employing the Kimura 2 substitution model with invariant sites. Node support was assessed after 1000 bootstrap pseudo-replicates. The GenBank accession numbers of analysed sequences are given in Additional file 2: Table S3.

## Results

### Prevalence of *Trichomonas* in columbids

**Fig. 1:**
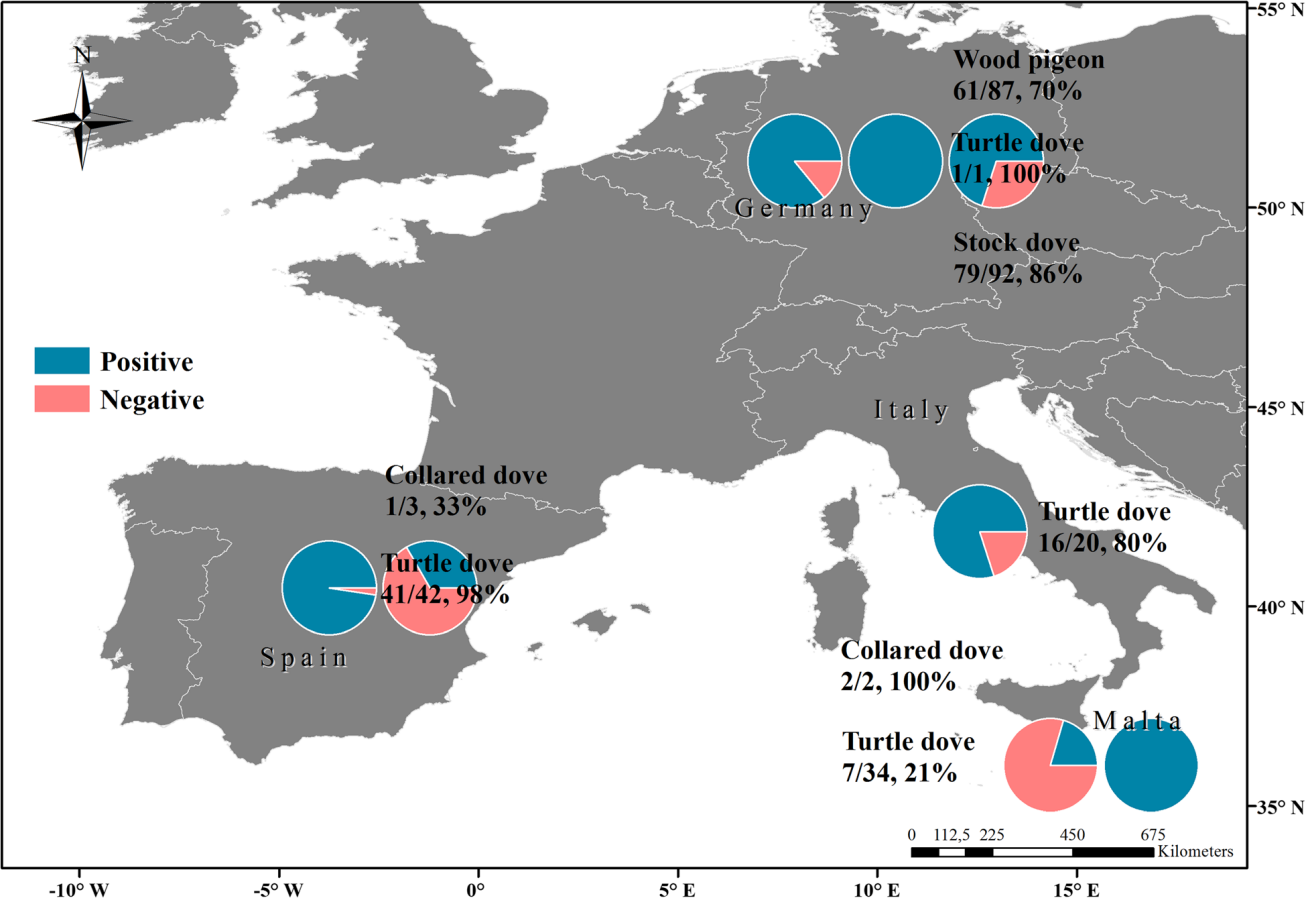
Map of sample sites and prevalence of *Trichomonas* in columbids from Germany, Italy and Spain. Next to the pie charts, the species name, the number of positive *Trichomonas* samples out of the sample size and percentages of positive *Trichomonas* samples within a species from one country are given. The results for wood pigeons arise from tissue samples. Furthermore, 27 samples of Maltese turtle doves were tissue samples. All of those were tested PCR-negative for *Trichomonas*

We found an overall prevalence of 74% across all 281 columbid samples with a significant difference between species (*χ* ^2^ = 91.023, *df* = 3, *P* = 0.007). Within swab samples, there was a total prevalence of 93% (*n* = 154) and within tissue samples a prevalence of 54% (*n* = 61) (Table 1). The majority of wood pigeons (*n* = 61; 70%) and stock doves (*n* = 79; 86%) were infected. From stock doves, 28 adults showed infection (30%), and 51 chicks were infected (55%). The first-year-old bird was not infected. Also, turtle doves (*n* = 65; 93%) showed high prevalence (Table 1). Additionally, turtle doves from different countries displayed high prevalence for every country (Fig. 1), including the one German turtle dove tested positive for *Trichomonas*. Only turtle doves from Malta had > 50% samples testing negative for *Trichomonas* (Fig. 1).

### Phylogenetic analysis of *Trichomonas* lineages

From 124 PCR products sequenced for the ITS/5.8S/ITS2 region, 84 were successfully assembled and used for further phylogenetic analysis (Table 1). Unfortunately, not all PCR products were able to assemble, due to their insufficient quality. The same applies to the PCR products sequenced for the Fe-hydrogenase region. Only three samples were sequenced successfully.

**Fig. 2:**
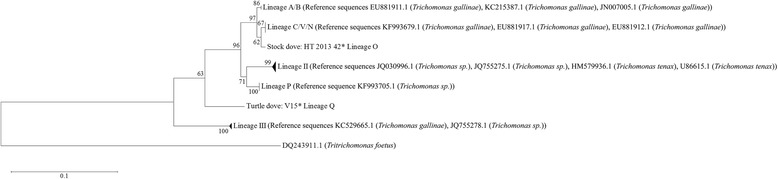
Phylogenetic analysis of the ITS1/5.8S/ITS2 ribosomal region of *Trichomonas* sp. Lineages found within this study together with the identical reference sequences from GenBank analysed using maximum likelihood. Reference sequences are labelled by GenBank accession number and *Trichomonas* species. References to GenBank accession numbers are as follows: JQ030996.1, JQ755275.1 and JQ755278.1 (Peters & Raidal, unpublished; samples from Australia), U86615.1 ([32]; sample from Switzerland), HM579936.1 ([57]; sample from France), KF993705.1 and KF993679.1 (Martínez-Herrero et al. unpublished; samples from Spain), EU881911.1, EU881912.1 and EU881917.1 ([12]; samples from Spain), KC215387.1 ([14]; sample from USA), JN007005.1 ([62]; sample from Austria), KC529665.1 ([42]; sample from UK), DQ243911.1 ([34]; sample from France). Asterisk marks the two single sequences and their sample names, which could not be linked to any of the previously described lineages

The phylogenetic tree for the ITS1/5.8S/ITS2 region contained seven different lineages (Fig. 2, Additional file 4: Figure S1). The lineage names were given according to first discoverers and where new lineages were found, the lineage names were labelled according to the nomenclature of Gerhold et al. [44]. Most samples clustered in lineages II [12] and C/V/N [42, 44], followed by lineage P (Table 2, Additional file 1: Table S1). Wood pigeons were predominantly infected by lineages II [12] and C/V/N [42, 44]. Turtle doves showed infections mainly caused by the P and III [42] lineage. Most of the Italian turtle dove samples belonged to the P and III [42] lineage (*n* = 4 for each lineage). One turtle dove sample from Italy could not be linked to a previously described *Trichomonas* lineage, but the closest GenBank match was KF993705.1 with 92% maximum identity and 98% query coverage (Table 2, Additional file 1: Table S1). Additionally, another stock dove sample could not be linked to a described lineage. Its closest GenBank match was EU881912.1 with 97% maximum identity and 96% query coverage (Table 2, Additional file 1: Table S1). The Spanish samples mainly belonged to the P and C/V/N [42, 44], lineages (*n* = 9 and *n* = 7, respectively). The Maltese samples occurred mainly in lineage P (*n* = 3).

**Table 2:** Summary of *Trichomonas* lineages found in this study compared to sequences described previously

Lineage	No. of infected birds	% infected birds	Species	No. of species	% species	Lineage name by Lennon et al. [12]	Lineage name by Gerhold et al. [44]	Lineage name by Chi et al. [42]
A/B_B_^a^	6	7.1	SD	4	25.0	3 (TD, WP) and 4 (WP)	A and B	A and B
			CD	2	100			
C/V/N_A_^b^	23	27.4	SD	4	25.0	1 (TD, WP)	C, D and E	C and V
			WP	10	43.5			
			TD	9	20.9			
O	1	1.2	SD	1	6.3	Not found	Not found	Not found
II	22	26.2	SD	3	18.8	2 (SD, TD, WP)	Not found	II
			WP	12	52.2			
			TD	7	16.3			
P	19	22.6	SD	2	12.5	Not found	Not found	Not found
			WP	1	4.4			
			TD	16	37.2			
Q	1	1.2	TD	1	2.3	Not found	Not found	Not found
III	12	14.3	SD	2	12.5	Not found	Not found	III
			TD	10	23.3			

*Notes*: For lineages obtained in our study and for the similar study by [12] we displayed the wild columbid host species as well as numbers and percentages of infected individuals. Furthermore, we give the numbers and percentages of lineages found in a species. Note, the percentages in a species (% species) were calculated according to the number of species infected by a certain lineage and divided through the absolute number of infected individuals of a species

*Abbreviations*: *SD* stock dove, *WP* wood pigeon, *TD* turtle dove, *CD* collared dove

^a^The lineage is synonymous with genotype B described by Sansano-Maestre et al. [14] and is therefore potentially pathogenic

^b^The lineage is synonymous with genotype A described by Sansano-Maestre et al. [14] and is therefore apparently non-pathogenic and widespread

Collared doves and stock doves were the only species infected by lineage A/B [44]. Besides, six out of seven *Trichomonas* lineages detected in this study occurred in stock doves. The four chicks from two stock dove nests were infected by different *Trichomonas* lineages. The first two siblings showed *Trichomonas* from lineage A/B [44]. The other two were infected by two different *Trichomonas* lineages (P and III [42]).

The samples sequenced for the Fe-hydrogenase gene clustered in sub-lineage P1 distinct to the reference sequences and sub-lineages (Additional file 5: Figure S2).

## Discussion

### Prevalence of *Trichomonas* in columbids

Stock doves, wood pigeons, collared doves and turtle doves in our study throughout a range of European sites showed a high prevalence of infection by *Trichomonas* sp. These results are in agreement with previous findings in the UK [12].

Although we only had a small sample size of collared doves, the prevalence seems high (67%). Additionally, if we separate the results by countries, Spanish collared doves suggest a much lower prevalence by *Trichomonas* sp. (33%) than in the UK (86%, see [12]) and lie closer to the prevalence shown in Iraq (10%, see [45]), but the samples from Malta showed a prevalence closer to the results shown in the UK (Fig. 1). Both compared studies used swab samples [12, 46] as we did in our survey, thus the differences cannot be linked to the different sample material. Furthermore, Al-Bakry [45] used a larger sample size of collared doves (*n* = 40) compared to Lennon et al. [12], who used seven individuals. Thus, the Iraqi results might be more reliable and a low *Trichomonas* prevalence in collared doves, as shown in the present study, may be more realistic. However, the small sample size in our study and Lennon et al. [12] should be interpreted carefully.

In our study, stock doves had a much higher prevalence than previously suggested (22–40% [12, 45]). Furthermore, chicks had higher prevalence of *Trichomonas* than adults, which is in agreement with Bunbury [46], who showed the negative impact of trichomonosis in Mauritian Pink pigeon chicks until an age of three months. In the present study, stock dove chicks were between five and 22 days old and therefore, within the most common time for infection with *Trichomonas* parasites in pigeon chicks [46].

In turtle doves, a high infection status of 67% was detected, which is 1/3 lower than previously observed in the UK (95%) [R. C. Thomas, unpublished data]. However, if we disregard the prevalence results of defrosted tissue samples from Maltese turtle doves, we would have a prevalence of 93% in turtle doves from Europe (Table 1), thus very similar to results from the UK.

On the other hand, our results revealed much higher prevalence for wood pigeons than previously suggested (47%, see [12]). However, it needs to be taken into account that the 70% prevalence of *Trichomonas* infection in German wood pigeons and the very low prevalence in turtle doves from Malta likely underestimates the true prevalence, because samples were not cultured directly, but analysed from tissues after freezing and defrosting. This might cause major differences in the detection rate, because of DNA degradation due to several freezing and thawing cycles, especially for Maltese samples during transportation [47–50]. Furthermore, Dunn et al. [51] showed the need to culture *Trichomonas* samples to reliably detect the infection. However, our results suggest high prevalence, at least for wood pigeons, but note that Maltese tissue samples were treated with a different *Taq* DNA Polymerase than wood pigeon samples. Thus, it might be the Dream*Taq* DNA Polymerase was less sensitive to DNA in thawed tissue samples.

Compared to other columbid species like mourning doves (*Zenaida macroura*), the overall prevalence of *Trichomonas* protozoan in the species studied here lies at the top of prevalence ranges. Mourning doves showed a very low prevalence of only 5.6% ranging from 4.4 to 10.6% [52]. In the endangered Mauritian pink pigeon the average prevalence of *Trichomonas* was 50% (ranging from 20 to 82%) [37], and therefore lower than in turtle doves, which are listed as a vulnerable species [28]. However, such prevalence has already been highlighted as a major threat to pink pigeons’ population recovery [37]. This might be caused by their small distribution range and population size, but also reveals the importance of gaining knowledge about parasite infection, particularly in recovering and declining birds. In the UK, trichomonosis has been indicated as a potential additive factor for the reported population decline of turtle doves since the 1970s [12]. Furthermore, Calderon et al. [53] already highlighted the decreasing effective population size of turtle doves, which makes them more vulnerable to threats. Thus, the present study suggests a potentially strong impact of *Trichomonas* on declining turtle doves across western and central Europe and likewise on recovering German stock dove populations.

Turtle doves are likely to be more dependent on anthropogenic food sources at feeding sites than in the 1960s, which was described in detail in the UK [54]. However, this circumstance might not only be limited to Great Britain, since it is linked to increased use of herbicides resulting from agricultural intensification, which has occurred throughout Europe [55, 56]. Furthermore, in the last century a change and intensification in forest management occurred as well [56], which led to a decreased number of natural breeding holes. That is why nowadays stock doves are largely dependent on artificial nest boxes in Germany [19, 56]. In Hesse, the stock dove population recovered locally, through provision of artificial nest boxes. However, besides increased food stress or insufficient natural breeding sites [29], turtle doves, stock doves, but also wood pigeons are migratory birds [19], which is why they may be more exposed to a wider range of parasites and pathogens of different bird species or populations (stop-overs at feeding and water sites) [15, 19, 21, 55]. Moreover, despite the existence of three main migratory flyways [18], European turtle doves show a lack of genetic structure [53]. That increases the probabilities for higher vulnerability and potential fast spread of infection among turtle dove populations.

Resident species, in contrast, may be less prone to infection, as shown for collared doves in Iraq [41] and Spain (this study, but see [12]). On the other hand, collared doves in Malta indicated a 100% *Trichomonas* prevalence.

As the disease occurs worldwide and is rapidly spreading, i.e. among wild finches [4], it seems likely that especially turtle doves and stock doves are also infected in other European countries. Regarding turtle doves, information on the disease in eastern Europe would be especially interesting, since, to our knowledge, no data on prevalence are available yet from the eastern European distribution range. Furthermore, the transmission may be reduced where no supplementary food is provided for endangered or vulnerable species and gamebirds [12].

### Phylogenetic relationships among *Trichomonas* lineages

Several attempts have been made to classify the genetic diversity of *Trichomonas* parasites in birds, with ensuing different nomenclatures (Table 2). Out of a total of seven genetic lineages found in the present study, three lineages were found in all columbid species we examined: lineages II [12], P and C/V/N [42, 44] (Fig. 2, Additional file 4: Figure S1). Of those, lineages II [12] and P belong to different *Trichomonas* species (*T. tenax* and *Trichomonas* sp., which grouped within *T. canistomae* [55]). However, species identification, based on morphological methods, may need to be revised and complemented as more genetic data are available. Since several *Trichomonas* species have been reported in birds based solely on morphology [12, 33, 43, 51,], the nomenclature for *Trichomonas* might also need revision as more molecular-based phylogenetic analyses are available. However, judging by the overall occurrence of these three lineages, our findings suggest a widespread distribution of those lineages across columbids [12, 33]. For instance, lineages II [12] and C/V/N [42, 44] were also predominant in species from the UK, Austria and the USA [12, 55]. Lineage II [12] even infected the same host species as shown in the UK [19] (Table 2).

Lineage C/V/N [42, 44] has also been described as “genotype A”, an apparently non-pathogenic *Trichomonas* lineage [19] with a global and frequent occurrence. It was previously found in turtle doves and wood pigeons [19], and we here additionally demonstrate infection in stock doves.

Regarding existing literature and previously described lineages, lineages O, P and Q might be newly detected lineages, because they were not described in previous studies [12, 22, 33, 42, 44, 57]. The samples from lineage P sequenced for the Fe-hydrogenase gene also clustered in a distinct and apparently new group of Fe-hydrogenase sub-lineage P1 (S4). Furthermore, lineages O and Q appear distinct to lineages A/B and C/V/N [42, 44], thus they may not be as common or widespread as lineages II, P and C/V/N [12, 42, 44], because they were only found in one German stock dove sample (O) and one Italian turtle dove sample (Q). Additionally, lineage III [42], found in stock doves and turtle doves, was identical to assigned reference sequences isolated from feral pigeons and was only found in Austria and the UK [42, 58]. Thus, we confirm its presence in turtle doves from Malta, Italy and Spain and in stock doves from Germany.

Only, lineage A/B [44] grouped with a potentially fatal lineage (genotype B) [12, 16, 19, 22], which was also responsible for the finch trichomonosis epizootic in the UK [22] (Table 2). This lineage, which is often lethal, has also been detected in turtle doves, wood pigeons and other non-passerines [12, 16, 22, 27] and we here confirm its presence in stock doves from Germany and resident collared doves from Malta. A fatal case of trichomonosis in a stock dove from Germany has been described previously [16] based on necrotic lesions and *Trichomonas* presence in microscopic analyses [16]. The lethal course of the disease hints to an infection by lineage A/B [44], although it was not genetically confirmed.

The lethal character of trichomonosis was described also in the Mauritian pink pigeon, a resident species on Mauritius similar to the collared doves on Comino (Malta) [52]. Thus, at least on Comino, the collared dove population might decrease in the future due to this potentially pathogenic lineage. Some years ago, the collared dove population already crashed due to an outbreak of a disease, which affected the region around the beak (B. Metzger, personal communication). No further description of the illness is known, but it is possible that it was trichomonosis.

Two genotypes of *T. gallinae* have previously been proposed to exhibit a differential in pathogenicity. Lineage C/V/N [42, 44] is synonymous with genotype A, described by Sansano-Maestre [19] as a wide-spread *Trichomonas* lineage, with mild or no pathogenicity. Lineage A/B [44] is synonymous with genotype B [19] described as possessing a more severe pathogenicity. Regarding all other lineages, pathogenicity has not been assessed. For this purpose, transmission experiments would be very helpful to see, if birds show signs of active trichomonosis and if they recover from infection and acquire possible immunity [25, 26].

### Phylogenetic analysis of *Trichomonas* lineages from stock dove siblings

To our knowledge, this is the first report of phylogenetic examination of *Trichomonas* from stock dove nestlings belonging to the same nest. Both findings (siblings), were infected by the same lineage as well as by different lineages; this can be explained by disease transmission *via* crop milk feeding, as both parents share chick feeding [19]. Thus, if both parents are infected by the same *Trichomonas* lineage, chicks receive the same *Trichomonas* pathogen. If both parents are infected by *Trichomonas* but carry different lineages, their chicks might be infected by different pathogens as well. Additionally, studies have shown [14, 59, 60], that individual birds may carry more than one *Trichomonas* lineage, so it is possible we only sequenced one strain when more than one was present. Here, we found a *T. gallinae* and *T. canistomae*-like lineage in two siblings of a stock dove nest. A coinfection of an apparently non-pathogenic *T. gallinae* (genotype A) [14] lineage plus a *T. tenax*-like strain was found previously in pigeons [60].

## Conclusions

Our results are in agreement with previous findings of geographically widespread *Trichomonas* lineages. We provide information about diverse lineages from Germany, Spain and Italy across different columbid species. Especially, lineages 2 [12], P and C/V/N [42, 44] were identified in all species and might, therefore, represent the most common *Trichomonas* lineages, at least in Columbiformes. However, only stock doves and collared doves from this study showed infections by the potentially pathogenic and often lethal *Trichomonas* lineages. Furthermore, we detected three newly discovered lineages (O, P and Q) and one additional sub-lineage based on the Fe-hydrogenase gene P1. Additionally, only one German stock dove sample was assigned to lineage O, and one Italian turtle dove sample was assigned to lineage Q. Due to the higher dependency on anthropogenic food sources [19, 54] and artificial nesting sites [19, 29] of turtle doves and stock doves as well as the migratory character of wood pigeons, turtle doves and stock doves these three species might have a higher risk to ingest *Trichomonas* protozoans of different lineages from various bird species and populations at shared feeding and water sites. This might be especially dangerous for recovering stock dove populations and declining turtle dove populations when parasites spread during times of food shortage or stress. Furthermore, it may be particularly worrying when stock doves or turtle doves get infected by potentially pathogenic lineages as we have shown here for stock doves from Germany and has already been shown for turtle doves in the UK [12]. The occurrence of potentially pathogenic lineages of *Trichomonas* in resident collared doves on Comino (Malta) might raise the concern for turtle doves even more since they share watering and feeding sites on the island. However, to estimate an impact on the population level, it would be very important to gain further knowledge about the prevalence of *Trichomonas* protozoans and the occurring lineages in turtle doves from Eastern European countries and to compare these data to turtle doves from Western Europe.

## Additional files

Additional file 1: Table S1. Sample names from different columbid hosts with their closest GenBank match for the ITS1-5.8S-ITS2 region, maximum identity and query coverage in % as well as the *Trichomonas* species of the GenBank match, the lineage, host and country in which the reference was found. (DOCX 25 kb)

Additional file 2: Table S3. The GenBank accession numbers (KX459439–KY675299) of the study sequences are listed in the table below. (DOCX 14 kb)

Additional file 3: Table S2. Sample names from different columbid hosts with their closest GenBank match for the Fe-hydrogenase region, maximum identity and query coverage in % as well as the parasite species of the reference. (DOCX 16 kb)

Additional file 4: Figure S1. Expanded phylogenetic tree including information about *Trichomonas* species and origin countries of reference sequences. Furthermore, the host species and sample ID of studied sequences are shown. (PDF 7856 kb)

Additional file 5: Figure S2. Phylogenetic tree based on the analysis of the Fe-hydrogenase gene of *Trichomonas gallinae*. This figure includes information about *Trichomonas* sub-lineages (A1, A1.1-A1.3, A2, C1-C4 and the newly detected sub-lineage P1). Furthermore, information about the origin countries of reference sequences is given, when information was available. Additionally, the host species and sample ID of studied sequences are shown. The break in the direction to sub-lineage P1 equals two substitutions. References to GenBank accession numbers are as follows: AF446077.1 [61], HG008115.1 [8], KC529660.1, KC529661.1, KC529662.1, KC529663.1, KC529664.1, KC962158.1 [42], JF681136.1 and JF681141.1 [22] and XM_001310179.1 [43]. (TIF 7369 kb)
