# Supplementary material for: High prevalence of Trichomonas gallinae in wild columbids across western and southern Europe
Source: Parasit Vectors. 2017 May 18;10:242. doi: 10.1186/s13071-017-2170-0 (PMC5437606; doi:10.1186/s13071-017-2170-0)
Supplement: Supplementary file 1 — Sample names from different columbid hosts with their closest GenBank match for the ITS1-5.8S-ITS2 region, maximum identity and query coverage in % as well as the Trichomonas species of the GenBank match, the lineage, host and country in which the reference was found. (DOCX 25 kb) [file 13071_2017_2170_MOESM1_ESM.docx]

***Trichomonas gallinae* in Westeuropean Wild Columbids: A Phylogenetic Analysis**

Melanie Marx^1^*, Gerald Reiner^2^, Hermann Willems^2^, Gregorio Rocha^3^, Klaus Hillerich^4^, Juan F. Masello^1^, Sylvia L. Mayr^2^, Sarah Moussa^1^, Jenny C. Dunn^5^, Rebecca C. Thomas^6^, Simon J. Goodman^6^, Keith C. Hamer^6^, Benjamin Metzger^7^, Jacopo G. Cecere^8^, Fernando Spina^8^, Steffen Koschkar^9^, Luciano Calderón^1^, Tanja Romeike^1^ and Petra Quillfeldt^1^

**Additional file 1**

**Table S1:** Sample names from different columbid hosts with their closest GenBank match for ITS1/5.8S/ITS2 region, maximum identity and query coverage in % as well as the *Trichomonas* species of the GenBank match, the lineage, host and country in which the reference was found.

| **Sample** | **Species** | **Country sample** | **Lineage** | **Closest GenBank match** | **Max ident [%]** | **Query coverage [%]** | ***Trichomonas* Species** | **Host** | **Country reference** |
| --- | --- | --- | --- | --- | --- | --- | --- | --- | --- |
| T-MA-6 | Turtle dove | Malta | P | KF993705.1 | 99 | 91 | *T.sp.* | *Streptopelia turtur* | Spain |
| T-MA-7 | Turtle dove | Malta | P | KF993705.1 | 96 | 100 | *T.sp.* | *Streptopelia turtur* | Spain |
| T-MA-8 | Turtle dove | Malta | III | KC529665.1 | 99 | 100 | *T.gallinae* | *pigeon* | UK |
| T-MA-9 | Turtle dove | Malta | P | KF993705.1 | 100 | 95 | *T.sp.* | *Streptopelia turtur* | Spain |
| T-MA-12 | Turtle dove | Malta | II | HM579936.1 | 98 | 90 | *T.tenax* | *Human* | France |
| T-MA-19 | Turtle dove | Malta | III | KC529665.1 | 99 | 99 | *T.gallinae* |  | UK |
| T-MA-22 | Collared dove | Malta | A/B | KC215387.1 | 100 | 99 | *T.gallinae* | *Patagioenas fasciata monilis* | USA |
| T-MA-23 | Collared dove | Malta | A/B | KC215387.1 | 100 | 99 | *T.gallinae* | *Patagioenas fasciata monilis* | USA |
| HT_2013_17 | Hohltaube | Germany | C/V/N | EU881912.1 | 99 | 96 | *T. gallinae* | *Columba livia* | Spain |
| HT_2013_35 | Hohltaube | Germany | A/B | EU881911.1 | 99 | 99 | *T. gallinae* | *Columba livia* | Spain |
| HT_2013_38 | Hohltaube | Germany | C/V/N | EU881912.1 | 99 | 99 | *T. gallinae* | *Columba livia* | Spain |
| HT_2013_42 | Hohltaube | Germany | O | EU881912.1 | 97 | 96 | *T. gallinae* | *Columba livia* | Spain |
| HT_2013_45 | Hohltaube | Germany | A/B | EU881911.1 | 99 | 99 | *T. gallinae* | *Columba livia* | Spain |
| HT_2013_46 | Hohltaube | Germany | A/B | KC215387.1 | 99 | 100 | *T. gallinae* | *Patagioenas fasciata monilis* | USA |
| HT_2013_47 | Hohltaube | Germany | A/B | KC215387.1 | 100 | 99 | *T. gallinae* | *Patagioenas fasciata monilis* | USA |
| HT_2013_53 | Hohltaube | Germany | C/V/N | KF993679.1 | 99 | 94 | *T. gallinae* | *Columba livia* | Spain |
| HT_2013_57 | Hohltaube | Germany | C/V/N | KF993679.1 | 99 | 95 | *T. gallinae* | *Columba livia* | Spain |
| HT_2014_5 | Hohltaube | Germany | III | KC529665.1 | 99 | 99 | *T. gallinae* | *Pigeon* | UK |
| HT_2014_14 | Hohltaube | Germany | II | U86615.1 | 99 | 98 | *T. tenax* | *Human* | Switzerland |
| HT_2014_16 | Hohltaube | Germany | III | KC529665.1 | 99 | 99 | *T. gallinae* | *Pigeon* | UK |
| HT_2014_17 | Hohltaube | Germany | P | KF993705.1 | 99 | 99 | *T. sp.* | *Streptopelia turtur* | Spain |
| HT_2014_22 | Hohltaube | Germany | II | HM579936.1 | 99 | 99 | *T. tenax* | *Human* | France |
| HT_2014_24 | Hohltaube | Germany | II | U86615.1 | 99 | 99 | *T. tenax* | *Human* | Switzerland |
| HT_2014_27 | Hohltaube | Germany | P | KF993705.1 | 99 | 99 | *T. sp.* | *Streptopelia turtur* | Spain |
| RT23_9_2013 | Wood pigeon | Germany | C/V/N | EU881912.1 | 100 | 100 | *T. gallinae* | *Columba livia* | Spain |
| RT_02 | Wood pigeon | Germany | II | U86615.1 | 99 | 95 | *T. tenax* | *Human* | Switzerland |
| RT_03 | Wood pigeon | Germany | II | U86615.1 | 99 | 98 | *T. tenax* | *Human* | Switzerland |
| RT_08 | Wood pigeon | Germany | C/V/N | EU881912.1 | 99 | 98 | *T. gallinae* | *Columba livia* | Spain |
| RT_09 | Wood pigeon | Germany | II | HM579936.1 | 99 | 100 | *T. tenax* | *Human* | France |
| RT_10 | Wood pigeon | Germany | II | U86615.1 | 99 | 98 | *T. tenax* | *Human* | Switzerland |
| RT_11 | Wood pigeon | Germany | II | U86615.1 | 99 | 98 | *T. tenax* | *Human* | Switzerland |
| RT_28 | Wood pigeon | Germany | II | JQ755275.1 | 99 | 98 | *T. sp.* | *Pigeon* | Australia |
| RT_31 | Wood pigeon | Germany | II | HM579936.1 | 99 | 99 | *T. tenax* | *Human* | France |
| RT_32 | Wood pigeon | Germany | II | HM579936.1 | 99 | 99 | *T. tenax* | *Human* | France |
| RT_42 | Wood pigeon | Germany | II | U86615.1 | 99 | 99 | *T. tenax* | *Human* | Switzerland |
| RT_45 | Wood pigeon | Germany | II | U86615.1 | 99 | 98 | *T. tenax* | *Human* | Switzerland |
| RT_46 | Wood pigeon | Germany | II | U86615.1 | 99 | 97 | *T. tenax* | *Human* | Switzerland |
| RT_47 | Wood pigeon | Germany | II | U86615.1 | 99 | 98 | *T. tenax* | *Human* | Switzerland |
| RTJ1 | Wood pigeon | Germany | P | KF993705.1 | 100 | 100 | *T. sp.* | *Streptopelia turtur* | Spain |
| RTJ3 | Wood pigeon | Germany | C/V/N | EU881912.1 | 100 | 100 | *T. gallinae* | *Columba livia* | Spain |
| RTNS1 | Wood pigeon | Germany | C/V/N | EU881912.1 | 99 | 100 | *T. gallinae* | *Columba livia* | Spain |
| RTNS2 | Wood pigeon | Germany | C/V/N | EU881912.1 | 100 | 100 | *T. gallinae* | *Columba livia* | Spain |
| RTNS5 | Wood pigeon | Germany | C/V/N | EU881912.1 | 100 | 100 | *T. gallinae* | *Columba livia* | Spain |
| RTNS6 | Wood pigeon | Germany | C/V/N | EU881912.1 | 99 | 99 | *T. gallinae* | *Columba livia* | Spain |
| RTNS7 | Wood pigeon | Germany | C/V/N | EU881912.1 | 99 | 100 | *T. gallinae* | *Columba livia* | Spain |
| WP4 | Wood pigeon | Germany | C/V/N | EU881912.1 | 99 | 100 | *T. gallinae* | *Columba livia* | Spain |
| WP6 | Wood pigeon | Germany | C/V/N | EU881912.1 | 100 | 100 | *T. gallinae* | *Columba livia* | Spain |
| TT-ES_1 | Turtle dove | Spain | II | HM579936.1 | 99 | 96 | *T. tenax* | *Human* | France |
| TT-ES_2 | Turtle dove | Spain | II | HM579936.1 | 99 | 99 | *T. tenax* | *Human* | France |
| TT-ES_3 | Turtle dove | Spain | P | KF993705.1 | 99 | 98 | *T. sp.* | *Streptopelia turtur* | Spain |
| TT-ES_4 | Turtle dove | Spain | P | KF993705.1 | 100 | 98 | *T. sp.* | *Streptopelia turtur* | Spain |
| TT-ES_5 | Turtle dove | Spain | P | KF993705.1 | 100 | 98 | *T. sp.* | *Streptopelia turtur* | Spain |
| TT-ES_6 | Turtle dove | Spain | P | KF993705.1 | 99 | 99 | *T. sp.* | *Streptopelia turtur* | Spain |
| TT-ES_7 | Turtle dove | Spain | III | KC529665.1 | 99 | 99 | *T. gallinae* | *Pigeon* | UK |
| TT-ES_11 | Turtle dove | Spain | C/V/N | EU881912.1 | 99 | 98 | *T. gallinae* | *Columba livia* | Spain |
| TT-ES_12 | Turtle dove | Spain | II | HM579936.1 | 99 | 98 | *T. tenax* | *Human* | France |
| TT-ES_13 | Turtle dove | Spain | P | KF993705.1 | 100 | 98 | *T. sp.* | *Streptopelia turtur* | Spain |
| TT-ES_14 | Turtle dove | Spain | P | KF993705.1 | 100 | 99 | *T. sp.* | *Streptopelia turtur* | Spain |
| TT-ES_15 | Turtle dove | Spain | C/V/N | EU881912.1 | 100 | 99 | *T. gallinae* | *Columba livia* | Spain |
| TT-ES_17 | Turtle dove | Spain | C/V/N | EU881912.1 | 100 | 99 | *T. gallinae* | *Columba livia* | Spain |
| TT-ES_18 | Turtle dove | Spain | P | KF993705.1 | 100 | 99 | *T. sp.* | *Streptopelia turtur* | Spain |
| TT-ES_21 | Turtle dove | Spain | P | KF993705.1 | 99 | 97 | *T. sp.* | *Streptopelia turtur* | Spain |
| TT-ES_26 | Turtle dove | Spain | C/V/N | EU881912.1 | 100 | 99 | *T. gallinae* | *Columba livia* | Spain |
| TT-ES_29 | Turtle dove | Spain | III | KC529665.1 | 99 | 99 | *T. gallinae* | *Pigeon* | UK |
| TT-ES-30 | Turtle dove | Spain | III | KC529665.1 | 99 | 98 | *T. gallinae* | *Pigeon* | UK |
| TT-ES_31 | Turtle dove | Spain | P | KF993705.1 | 100 | 98 | *T. sp.* | *Streptopelia turtur* | Spain |
| TT-ES_34 | Turtle dove | Spain | C/V/N | EU881912.1 | 99 | 100 | *T. gallinae* | *Columba livia* | Spain |
| TT-ES_36 | Turtle dove | Spain | C/V/N | EU881912.1 | 100 | 100 | *T. gallinae* | *Columba livia* | Spain |
| TT-ES_39 | Turtle dove | Spain | C/V/N | EU881912.1 | 99 | 99 | *T. gallinae* | *Columba livia* | Spain |
| TT-ES_40 | Turtle dove | Spain | III | JQ755278.1 | 99 | 99 | *T. sp.* | *Pigeon* | Australia |
| V2 | Turtle dove | Italy | II | U86615.1 | 99 | 99 | *T. tenax* | *Human* | Switzerland |
| V4 | Turtle dove | Italy | P | KF993705.1 | 100 | 99 | *T. sp.* | *Streptopelia turtur* | Spain |
| V5 | Turtle dove | Italy | III | KC529665.1 | 99 | 100 | *T. gallinae* | *Pigeon* | UK |
| V7 | Turtle dove | Italy | II | HM579936.1 | 99 | 99 | *T. tenax* | *Human* | France |
| V8 | Turtle dove | Italy | C/V/N | EU881912.1 | 100 | 99 | *T. gallinae* | *Columba livia* | Spain |
| V11 | Turtle dove | Italy | III | KC529665.1 | 99 | 100 | *T. gallinae* | *Pigeon* | UK |
| V12 | Turtle dove | Italy | III | KC529665.1 | 99 | 100 | *T. gallinae* | *Pigeon* | UK |
| V13 | Turtle dove | Italy | P | KF993705.1 | 100 | 100 | *T. sp.* | *Streptopelia turtur* | Spain |
| V14 | Turtle dove | Italy | P | KF993705.1 | 100 | 99 | *T. sp.* | *Streptopelia turtur* | Spain |
| V15 | Turtle dove | Italy | Q | KF993705.1 | 92 | 98 | *T. sp.* | *Streptopelia turtur* | Spain |
| V17 | Turtle dove | Italy | II | HM579936.1 | 99 | 100 | *T. tenax* | *Human* | France |
| V18 | Turtle dove | Italy | P | KF993705.1 | 100 | 100 | *T. sp.* | *Streptopelia turtur* | Spain |
| V19 | Turtle dove | Italy | C/V/N | EU881912.1 | 99 | 100 | *T. gallinae* | *Columba livia* | Spain |
| V20 | Turtle dove | Italy | III | KC529665.1 | 99 | 99 | *T. gallinae* | *Pigeon* | UK |
