# Supplementary material for: High prevalence of Trichomonas gallinae in wild columbids across western and southern Europe
Source: Parasit Vectors. 2017 May 18;10:242. doi: 10.1186/s13071-017-2170-0 (PMC5437606; doi:10.1186/s13071-017-2170-0)
Supplement: Supplementary file 2 — The GenBank accession numbers (KX459439–KY675299) of the study sequences are listed in the table below. (DOCX 14 kb) [file 13071_2017_2170_MOESM2_ESM.docx]

***Trichomonas gallinae* in Westeuropean Wild Columbids: A Phylogenetic Analysis**

Melanie Marx^1^*, Gerald Reiner^2^, Hermann Willems^2^, Gregorio Rocha^3^, Klaus Hillerich^4^, Juan F. Masello^1^, Sylvia L. Mayr^2^, Sarah Moussa^1^, Jenny C. Dunn^5^, Rebecca C. Thomas^6^, Simon J. Goodman^6^, Keith C. Hamer^6^, Benjamin Metzger^7^, Jacopo G. Cecere^8^, Fernando Spina^8^, Steffen Koschkar^9^, Luciano Calderón^1^, Tanja Romeike^1^ and Petra Quillfeldt^1^

**Additional file 2**

**Table S3:** The GenBank accession numbers (KX459439 – KY675299) of the study sequences are listed in the table below.

| Sample | ITS or FeDH | GenBank Accession number |
| --- | --- | --- |
| HT_2013_17 | ITS | KX459439 |
| HT_2013_35 | ITS | KX459440 |
| HT_2013_38 | ITS | KX459441 |
| HT_2013_42 | ITS | KX459442 |
| HT_2013_45 | ITS | KX459443 |
| HT_2013_46 | ITS | KX459444 |
| HT_2013_47 | ITS | KX459445 |
| HT_2013_53 | ITS | KX459446 |
| HT_2013_57 | ITS | KX459447 |
| HT_2014_5 | ITS | KX459448 |
| HT_2014_14 | ITS | KX459449 |
| HT_2014_16 | ITS | KX459450 |
| HT_2014_17 | ITS | KX459451 |
| HT_2014_22 | ITS | KX459452 |
| HT_2014_24 | ITS | KX459453 |
| HT_2014_27 | ITS | KX459454 |
| RT23_9_2013 | ITS | KX459455 |
| RT_02 | ITS | KX459456 |
| RT_03 | ITS | KX459457 |
| RT_08 | ITS | KX459458 |
| RT_09 | ITS | KX459459 |
| RT_10 | ITS | KX459460 |
| RT_11 | ITS | KX459461 |
| RT_28 | ITS | KX459462 |
| RT_31 | ITS | KX459463 |
| RT_32 | ITS | KX459464 |
| RT_42 | ITS | KX459465 |
| RT_45 | ITS | KX459466 |
| RT_46 | ITS | KX459467 |
| RT_47 | ITS | KX459468 |
| RTJ1 | ITS | KX459469 |
| RTJ3 | ITS | KX459470 |
| RTNS1 | ITS | KX459471 |
| RTNS2 | ITS | KX459472 |
| RTNS5 | ITS | KX459473 |
| RTNS6 | ITS | KX459474 |
| RTNS7 | ITS | KX459475 |
| WP4 | ITS | KX459476 |
| WP6 | ITS | KX459477 |
| TT-ES_1 | ITS | KX459478 |
| TT-ES_2 | ITS | KX459479 |
| TT-ES_3 | ITS | KX459480 |
| TT-ES_4 | ITS | KX459481 |
| TT-ES_5 | ITS | KX459482 |
| TT-ES_6 | ITS | KX459483 |
| TT-ES_7 | ITS | KX459484 |
| TT-ES_11 | ITS | KX459485 |
| TT-ES_12 | ITS | KX459486 |
| TT-ES_13 | ITS | KX459487 |
| TT-ES_14 | ITS | KX459488 |
| TT-ES_15 | ITS | KX459489 |
| TT-ES_17 | ITS | KX459490 |
| TT-ES_18 | ITS | KX459491 |
| TT-ES_21 | ITS | KX459492 |
| TT-ES_26 | ITS | KX459493 |
| TT-ES_29 | ITS | KX459494 |
| TT-ES-30 | ITS | KX459495 |
| TT-ES_31 | ITS | KX459496 |
| TT-ES_34 | ITS | KX459497 |
| TT-ES_36 | ITS | KX459498 |
| TT-ES_39 | ITS | KX459499 |
| TT-ES_40 | ITS | KX459500 |
| V2 | ITS | KX459501 |
| V4 | ITS | KX459502 |
| V5 | ITS | KX459503 |
| V7 | ITS | KX459504 |
| V8 | ITS | KX459505 |
| V11 | ITS | KX459506 |
| V12 | ITS | KX459507 |
| V13 | ITS | KX459508 |
| V14 | ITS | KX459509 |
| V15 | ITS | KX459510 |
| V17 | ITS | KX459511 |
| V18 | ITS | KX459512 |
| V19 | ITS | KX459513 |
| V20 | ITS | KX459514 |
| T-MA-6 | ITS | KX844984 |
| T-MA-7 | ITS | KX844985 |
| T-MA-8 | ITS | KX844986 |
| T-MA-9 | ITS | KX844987 |
| T-MA-12 | ITS | KX844988 |
| T-MA-19 | ITS | KX844989 |
| T-MA-22 | ITS | KX844990 |
| T-MA-23 | ITS | KX844991 |
| V14 | FeDH | KY675297 |
| V18 | FeDH | KY675298 |
| T-MA-6 | FeDH | KY675299 |
