# Supplementary material for: High prevalence of Trichomonas gallinae in wild columbids across western and southern Europe
Source: Parasit Vectors. 2017 May 18;10:242. doi: 10.1186/s13071-017-2170-0 (PMC5437606; doi:10.1186/s13071-017-2170-0)
Supplement: Supplementary file 3 — Sample names from different columbid hosts with their closest GenBank match for the Fe-hydrogenase region, maximum identity and query coverage in % as well as the parasite species of the reference. (DOCX 16 kb) [file 13071_2017_2170_MOESM3_ESM.docx]

***Trichomonas gallinae* in Westeuropean Wild Columbids: A Phylogenetic Analysis**

Melanie Marx^1^*, Gerald Reiner^2^, Hermann Willems^2^, Gregorio Rocha^3^, Klaus Hillerich^4^, Juan F. Masello^1^, Sylvia L. Mayr^2^, Sarah Moussa^1^, Jenny C. Dunn^5^, Rebecca C. Thomas^6^, Simon J. Goodman^6^, Keith C. Hamer^6^, Benjamin Metzger^7^, Jacopo G. Cecere^8^, Fernando Spina^8^, Steffen Koschkar^9^, Luciano Calderón^1^, Tanja Romeike^1^ and Petra Quillfeldt^1^

**Additional file 3**

**Table S2:** Sample names from different columbid hosts with their closest GenBank match for Fe-hydrogenase region, maximum identity and query coverage in % as well as the parasite species of the reference.

| **Sample** | **Host** | **Closest GenBank match** | **Max ident [%]** | | **Query coverage [%]** | **Parasite Species** |
| --- | --- | --- | --- | --- | --- | --- |
| T-MA-6 | Turtle dove | AF446077.1 | 94 | 99 | | *Trichomonas gallinae* |
| V14 | Turtle dove | AF446077.1 | 94 | 99 | | *Trichomonas gallinae* |
| V18 | Turtle dove | AF446077.1 | 94 | 99 | | *Trichomonas gallinae* |
