# Supplementary figures and images for: High prevalence of Trichomonas gallinae in wild columbids across western and southern Europe
Source: Parasit Vectors. 2017 May 18;10:242. doi: 10.1186/s13071-017-2170-0 (PMC5437606; doi:10.1186/s13071-017-2170-0)

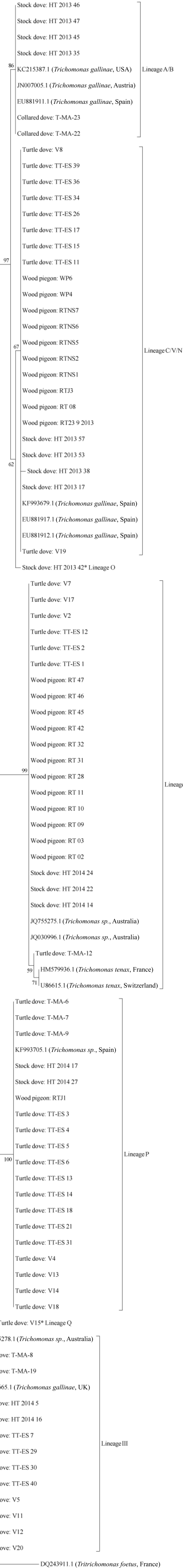

Supplement: Supplementary file 4 — Expanded phylogenetic tree including information about Trichomonas species and origin countries of reference sequences. Furthermore, the host species and sample ID of studied sequences are shown. (PDF 7856 kb) [file 13071_2017_2170_MOESM4_ESM.pdf]

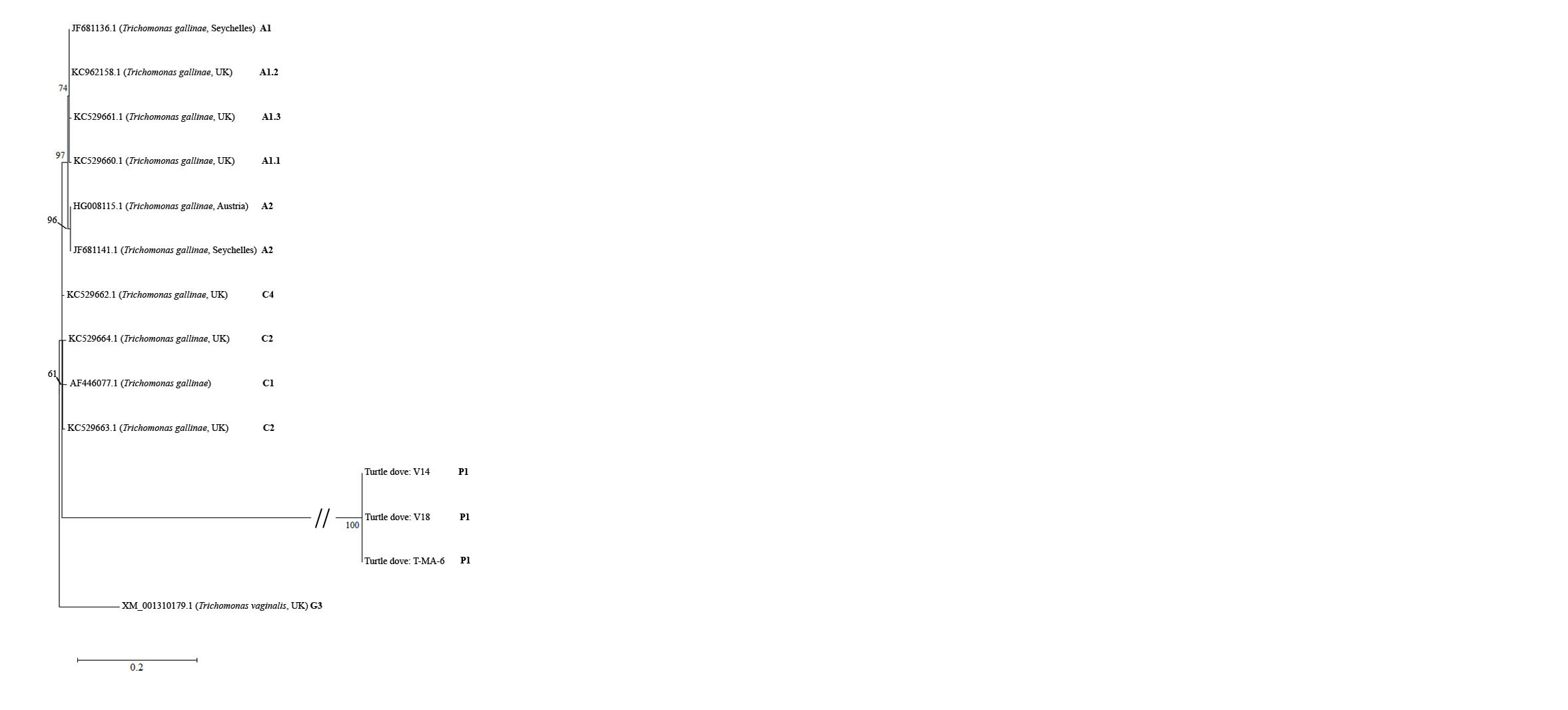

Supplement: Supplementary file 5 — Phylogenetic tree based on the analysis of the Fe-hydrogenase gene of Trichomonas gallinae. This figure includes information about Trichomonas sub-lineages (A1, A1.1-A1.3, A2, C1-C4 and the newly detected sub-lineage P1). Furthermore, information about the origin countries of reference sequences is given, when information was available. Additionally, the host species and sample ID of studied sequences are shown. The break in the direction to sub-lineage P1 equals two substitutions. References to GenBank accession numbers are as follows: AF446077.1 [61], HG008115.1 [8], KC529660.1, KC529661.1, KC529662.1, KC529663.1, KC529664.1, KC962158.1 [42], JF681136.1 and JF681141.1 [22] and XM_001310179.1 [43]. (TIF 7369 kb) [file 13071_2017_2170_MOESM5_ESM.tif]
